# How the vestibular system interacts with somatosensory perception: A sham-controlled study with galvanic vestibular stimulation

**DOI:** 10.1016/j.neulet.2013.06.046

**Published:** 2013-08-29

**Authors:** Elisa R. Ferrè, Brian L. Day, Gabriella Bottini, Patrick Haggard

**Affiliations:** a Institute of Cognitive Neuroscience, University College London, London, UK; b Sobell Department of Motor Neuroscience and Movement Disorders, UCL Institute of Neurology, London, UK; c Cognitive Neuropsychology Centre, Niguarda Ca’ Granda Hospital, MIlan, Italy; d Department of Brain and Behavioral Sciences, University of Pavia, Pavia, Italy

**Keywords:** CVS, caloric vestibular stimulation, GVS, galvanic vestibular stimulation, EPSPs, excitatory postsynaptic potentials, OP, parietal operculum, SII, secondary somatosensory cortex, PIVC, parieto insular vestibular cortex, SSDT, somatosensory signal detection task, Galvanic vestibular stimulation, Vestibular system, Tactile perception, Multisensory integration

## Abstract

The vestibular system has widespread interactions with other sensory modalities. Here we investigate whether vestibular stimulation modulates somatosensory function, by assessing the ability to detect faint tactile stimuli to the fingertips of the left and right hand with or without galvanic vestibular stimulation (GVS). We found that left anodal and right cathodal GVS, significantly enhanced sensitivity to mild shocks on either hand, without affecting response bias. There was no such effect with either right anodal and left cathodal GVS or sham stimulation. Further, the enhancement of somatosensory sensitivity following GVS does not strongly depend on the duration of GVS, or the interval between GVS and tactile stimulation. Vestibular inputs reach the somatosensory cortex, increasing the sensitivity of perceptual circuitry.

## Highlights

• Left anodal galvanic vestibular stimulation increased tactile sensitivity.
• No effects induced by sham stimulation or right anodal galvanic vestibular stimulation.
• Even brief (100 ms) pulses of vestibular stimulation enhanced somatosensory detection.
• Vestibular projections in the right hemisphere modulates somatosensory processing.

## 1 Introduction

The cortical vestibular system is strongly integrated with other sensory modalities, including somatosensory processing [14]. We previously reported that cold caloric vestibular stimulation (CVS) increases tactile sensitivity on the fingers of both hands [4,6]. Additionally, somatosensory potentials evoked by median nerve stimulation are modulated by CVS [5]. In particular, CVS selectively enhanced the N80 component recorded over both ipsilateral and contralateral somatosensory areas, without significantly affecting earlier or later components. Interestingly, the N80 component has been localised to the parietal operculum (OP) [10], which includes the human homologue of the monkey parieto insular vestibular cortex (PIVC) [3,14,22], and is thus a prime neuroanatomical candidate for vestibular-somatosensory convergence [5].

However, CVS has important methodological limitations [15]. During CVS participant's ear is irrigated with cold water for few seconds. This technique does not permit a complete control of the parameters of the stimulation, for example the exact volume of water going into the external ear canal and the precise timing of the stimulation of the vestibular organs. Moreover, non-vestibular contributions to CVS-induced modulation of somatosensory processing, for example due to the cold sensation in the outer ear, cannot be ruled out, because of the absence of reliable sham stimulation.

Here the vestibular modulation of somatosensory perception is investigated using a well-controlled, quantitative method for activating the vestibular cortical projections. Galvanic vestibular stimulation (GVS) is a non-invasive technique [19] that involves a weak direct current passing between surface electrodes placed on the mastoid behind the ear [8]. GVS modulates the firing rate of vestibular afferents with perilymphatic cathodal currents causing an increase in firing rate and anodal currents causing a decrease [8]. Bipolar binaural GVS evokes a net pattern of firing across both vestibular organs that mimics a head motion in space [9]. Crucially, the polarity of stimulation can be reversed as part of the experimental procedure, producing opposite effects on firing rate in the two vestibular organs, and thus reversing of direction of the apparent head motion. Moreover, placing the GVS electrodes away from the mastoids allows a sham stimulation, producing the same skin sensations under the electrodes as real GVS, but without stimulation of the vestibular organs.

In the present study, we assessed effects of vestibular inputs on somatosensory perception by administering different GVS polarities and a sham condition (Experiment 1). We also explored the time-course of the vestibular-somatosensory interaction (Experiment 2).

## 2 Experiment 1: specificity of vestibular-somatosensory interaction

### 2.1 Participants

Twelve naïve paid participants volunteered in Experiment 1a (8 male, ages: 20–32 years, mean ± SD: 24.41 ± 3.94 years), in Experiment 1b (10 male, ages: 20–32 years, mean ± SD: 22.91 ± 3.80 years), and in Experiment 1c (10 male, ages: 20–32 years, mean ± SD: 22.91 ± 3.80 years). Six of those who participated in Experiment 1a also participated in Experiment 1b and Experiment 1c. All participants were right-handed [17] with no history of neurological disorders. The experimental protocol was approved by University College London research ethics committee.

### 2.2 Galvanic vestibular stimulation procedure

GVS was applied in bipolar configuration by a custom-built constant-current stimulator (Good Vibrations Engineering Ltd., Nobleton, Ontario, Canada) used to deliver a boxcar pulse of 1 mA (duration is given below for each experiment, see Sections 2.3 and 3.2). Carbon rubber electrodes (area 10 cm^2^) coated with electrode gel were placed binaurally over the mastoid processes and fixed in place with adhesive tape. Left anodal and right cathodal configuration was named ‘LGVS’ (Experiment 1a). The inverse polarity, namely left cathodal and right anodal configuration, was named ‘RGVS’ (Experiment 1b). Sham stimulation was applied in which the electrodes were placed on the left and right side of the neck (about 5 cm below the GVS electrodes) using left anodal and right cathodal configuration (Experiment 1c). This causes a similar tingling skin sensation to real GVS, so it functions as a sham control for non-vestibular effects. Such non-vestibular effects could include a direct somato-somatosensory interaction between the skin sensations generated by the GVS electrodes and by the finger electrodes, and also more general factors such as the knowledge that an unusual stimulation is occurring.

### 2.3 Somatosensory signal detection task (SSDT)

Participants performed a somatosensory signal detection task (SSDT) during LGVS (Experiment 1a), RGVS (Experiment 1b) and sham stimulation (Experiment 1c). The methods closely followed a previous study [4]. SSDT was administered using a repeated measure design with stimulation (off-stimulation vs on-stimulation), side of tactile stimulation (left finger vs right finger) as within-subject variables.

Tactile stimulation was provided by a custom-built electrical stimulator, whose current-level and pulse duration were controlled by a computer. Tactile stimuli were delivered via 4 mm diameter concentric electrodes [11] attached to the index fingertips by surgical tape. A staircase procedure [13] was used to estimate the tactile threshold and this value was used to determine the intensity of the tactile stimuli during the SSDT. Our design factorially combined GVS and tactile stimulation conditions. SSDT consisted of sixty trials at current levels slightly below estimated tactile threshold (−10%) and 60 catch trials. We also delivered 20 trials at current levels clearly above tactile detection (+10%). These above-threshold trials were intended to remind participants of the nature of the tactile signal being detected, and were not analysed further [4]. Trials were delivered both in an on-stimulation condition, and an off-stimulation condition in which the vestibular/sham current was zero. All combinations of GVS and tactile stimulation were randomised anew for each experiment and each participant.

The beginning of each trial was signalled by an auditory tone. For on-stimulation trials, vestibular/sham stimulation was delivered after a variable interval between 250 ms and 500 ms from the acoustic sound. Vestibular/sham stimulation was followed by 1000 ms of delay and then the cutaneous shock, if present, was administered. A different tone indicated the end of the trial after 500 ms of delay from the cutaneous shock. In each on-stimulation trial the overall duration of vestibular/sham stimulation was 1500 ms. Participants were required to indicate whether or not they felt the tactile stimulus. Off-stimulation trials had an identical timing, but no actual vestibular/sham stimulation current.

In Experiment 1a SSDT was performed in different body postures. In one condition, participants were asked to sit upright with a normal head posture. In a second condition, participants sat with the hips and neck flexed, in a head-down posture. This is known to maximise the effect of GVS by aligning Reid's plane with the vertical plane [2]. Experiment 1b and Experiment 1c were performed with head down postures.

The data from catch trials and from trials with intensity just below threshold were analysed using signal detection analysis [16]. The *d*′ measures of sensitivity, and the *C* measure of response bias were calculated for each participant in each condition. The same false alarm rate was used for both left and right fingers, so the *d*′ values for the two fingers are not fully independent, since they both include this common term.

### 2.4 Results

#### 2.4.1 Experiment 1a: LGVS

**Fig. 1:**
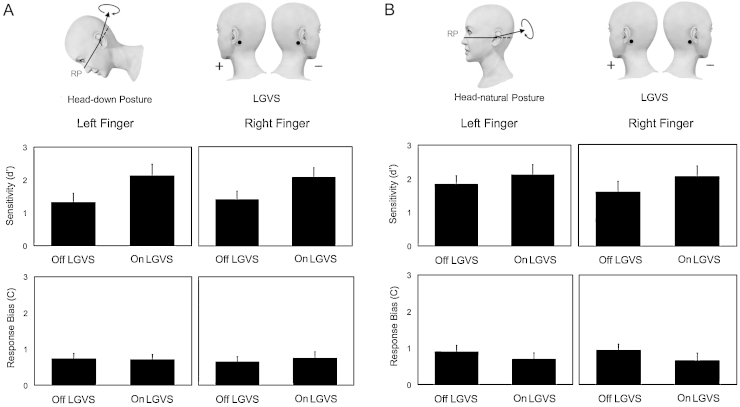
Effects of LGVS on somatosensory signal detection. (A) Perceptual sensitivity (*d*′) and response bias (*C*) values as a function of each condition (error bars indicate standard error of the mean) in head-down posture and (B) in head natural posture.

SSDT estimates of perceptual sensitivity (*d*′) and response bias (*C*) were analysed using 2 × 2 × 2 repeated measure ANOVA with factors of Stimulation (on-stimulation vs off-stimulation), Side of tactile stimulation (left hand vs right hand) and Body Posture (head-down vs head-natural) (Fig. 1).

Analysis of *d*′ showed a significant effect of Stimulation (*F*_(1,11)_ = 5.020, *p* = 0.047), with better tactile sensitivity when GVS was on than when it was off. There was no effect of Side (*F*_(1,11)_ = 0.102, *p* = 0.755) and no effect of Head Posture (*F*_(1,11)_ = 1.245, *p* = 0.288). No interactions between factors were significant (all *p* > 0.05). *C* values showed no significant main effect of Stimulation (*F*_(1,11)_ = 0.487, *p* = 0.500), or Side (*F*_(1,11)_ = 0.017, *p* = 0.898) or Head Posture (*F*_(1,11)_ = 2.207, *p* = 0.165). A significant interaction between Stimulation and Head Posture was found (*F*_(1,11)_ = 10.249, *p* = 0.008). This interaction was not predicted, but is reported here for completeness. Simple effects analysis was used to explore this interaction, holding the level of each factor constant and investigating the effects of the other factor. Thus, there was a significant difference between head-down posture and natural head posture (*t*_(11)_ = −3.235, *p* = 0.008) for the off-stimulation condition, but not for the on-stimulation condition (*p* > 0.05). No other significant comparisons were found.

#### 2.4.2 Experiment 1b: RGVS

**Fig. 2:**
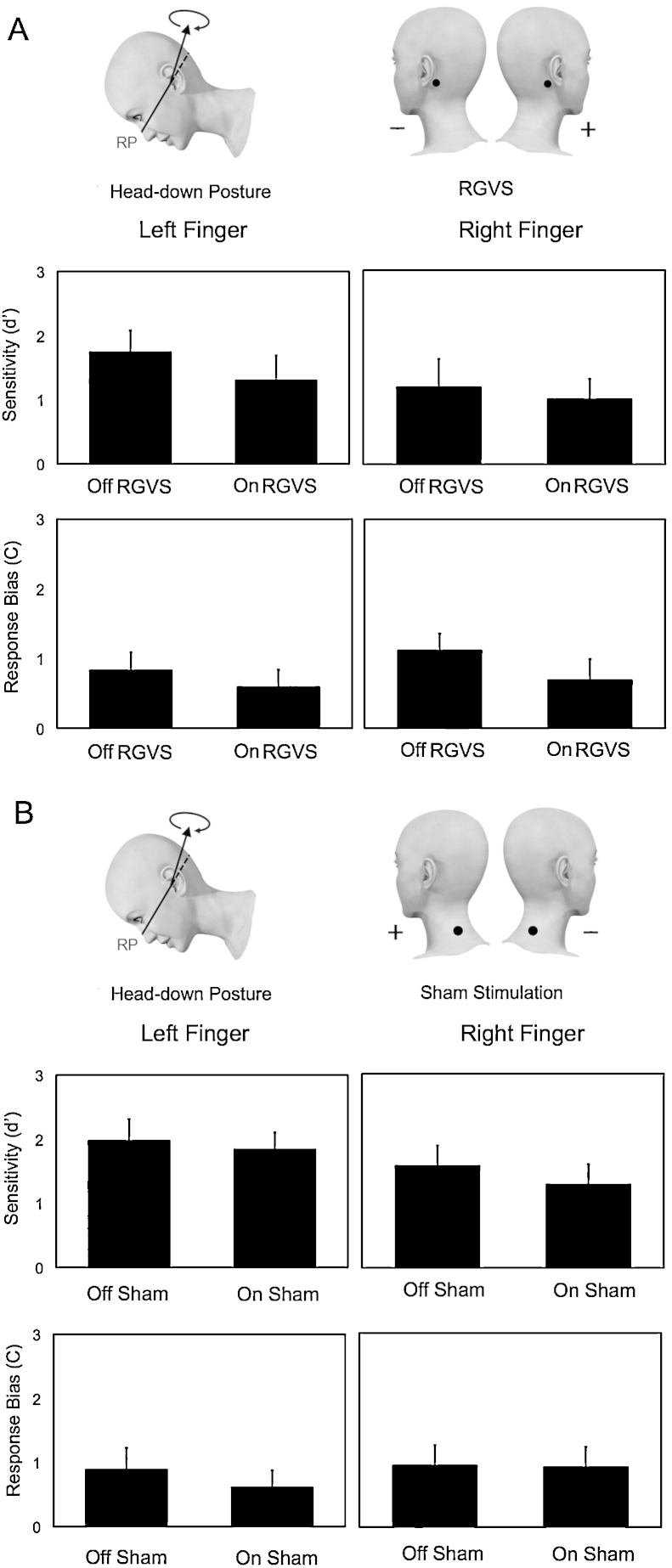
Effects of RGVS and sham stimulation on somatosensory signal detection. Perceptual sensitivity (*d*′) and response bias (*C*) values as a function of each condition (error bars indicate standard error of the mean) during (A) RGVS and (B) sham stimulation.

A 2 × 2 repeated measures ANOVA with factors of Stimulation (on-stimulation vs off-stimulation) and Side (left hand vs right hand) was applied to SSDT estimates of perceptual sensitivity (*d*′) and response bias (*C*) (Fig. 2A).

Analysis of *d*′ showed no significant effect of Stimulation (*F*_(1,11)_ = 2.345, *p* = 0.154) or Side (*F*_(1,11)_ = 3.047, *p* = 0.109) and no interaction between factors (*F*_(1,11)_ = 0.839, *p* = 0.379). *C* values showed no significant main effect of Stimulation (*F*_(1,11)_ = 4.052, *p* = 0.069), or Side (*F*_(1,11)_ = 2.729, *p* = 0.127) or interaction between Stimulation and Side (*F*_(1,11)_ = 1.153, *p* = 0.306).

#### 2.4.3 Experiment 1c: sham stimulation

Somatosensory signal detection task estimates of perceptual sensitivity (*d*′) and response bias (*C*) were analysed using 2 × 2 repeated measure ANOVA with factors of Stimulation (on-stimulation vs off-stimulation) and Side (left hand vs right hand) (Fig. 2B).

No significant effect of Stimulation (*F*_(1,11)_ = 1.015, *p* = 0.335) or Side (*F*_(1,11)_ = 3.963, *p* = 0.072) and interaction between factors (*F*_(1,11)_ = 1.225, *p* = 0.292) were found in *d*′ data. *C* values showed a significant main effect of Stimulation (*F*_(1,11)_ = 5.212, *p* = 0.043). No significant main effect of Side (*F*_(1,11)_ = 3.811, *p* = 0.077) and interaction between factors (*F*_(1,11)_ = 0.978, *p* = 0.344) were found.

#### 2.4.4 Between-experiments comparison: LGVS selectively increased tactile sensitivity

To investigate whether the enhancement of tactile sensitivity observed in Experiment 1a was specific for LGVS, we directly compared our three experimental conditions (Experiment 1a, Experiment 1b and Experiment 1c). Since Experiment 1b and Experiment 1c have been performed in head down posture, only the data in the head down posture condition of Experiment 1a were included in this analysis. The number of trials used as a basis for statistical calculations was, however, the same across all the experiments. SSDT estimates of perceptual sensitivity (*d*′) and response bias (*C*) were analysed using a 2 × 2 ANOVA with Stimulation (on-stimulation vs off-stimulation) and Side of tactile stimulation (left hand vs right hand) as within subjects factors and Condition (LGVS, RGVS and sham stimulation) as between subject variable. As noted above, some participants in fact performed more than one experiment. Within this mixed old-and-new sample, a between-subjects statistical analysis was used, to ensure a conservative test rather than an excessively liberal one.

Analysis of *d*′ showed a significant main effect of Side (*F*_(1,33)_ = 4.972, *p* = 0.033) and a significant interaction between Stimulation and Condition (*F*_(2,33)_ = 5.995, *p* = 0.006). No other main effects or interactions were significant (*p* > 0.05). This interaction was further explored by comparing the effect induced by the stimulation, measured as the difference between on-stimulation and off-stimulation, across LGVS, RGVS and sham stimulation. Independent *t*-tests showed a significant difference between LGVS and RGVS conditions (*t*_(22)_ = 2.705, *p* = 0.013) and between LGVS and sham stimulation conditions (*t*_(22)_ = 3.048, *p* = 0.006). Conversely, the comparison between RGVS and sham stimulation conditions was not significant (*t*_(22)_ = 0.326, *p* = 0.747).

*C* values showed a significant main effect of Side (*F*_(1,33)_ = 4.633, *p* = 0.039) and Stimulation (*F*_(1,33)_ = 4.225, *p* = 0.048). No significant interactions between factors were found (*p* > 0.05). Thus, delivering GVS modulates the response bias, making participants more liberal in reporting the tactile stimulus as present. Importantly, this shift in the response bias is present across experimental conditions, including sham, and does not affecting tactile sensitivity estimates.

### 2.5 Discussion

The main result of Experiment 1a was a significant modulation of tactile sensitivity induced by LGVS. This increase appeared to be bilateral, since we found no evidence for a difference depending on whether the left or right hand received tactile stimulation on either sensitivity or response bias. A laterality effect of this kind might have been expected given the strong leftward shift of attention produced by vestibular stimulation in patients [18,20,21]. We found no effect of head posture on GVS modulation of somatosensory perception. This may be surprising, given that Day et al. [2] reported an increase in perception of virtual head rotation in the horizontal plane (i.e. left and right) during GVS in head-down posture. Some changes in the response bias were elicited by head posture, making participants more liberal in responding ‘yes’ when they were in the head down posture. However, these changes were present in the baseline (off-stimulation condition), so are unrelated to GVS.

In Experiment 1b, no effects induced by RGVS on tactile sensitivity or response bias were found. Similar results were found with sham stimulation in Experiment 1c. However, sham stimulation did influence response bias making participants slightly more liberal. Importantly the numerical effects induced by both RGVS and sham stimulation on sensitivity were in the opposite direction to those found with LGVS. This observation is further supported by the difference between LGVS and RGVS and between LGVS and sham stimulation in a between-experiments comparison. Conversely no differences were found between RGVS and sham stimulation. Crucially, non-specific alerting cues such as skin tingling, or the knowledge that stimulation will occur, seem, if anything, to impair the sensitivity of tactile detection on the fingertips, while LGVS enhances it.

## 3 Experiment 2: Time and dose dependent investigation of vestibular-somatosensory interaction

### 3.1 Participants

Twenty naïve right-handed participants volunteered in this experiment (14 male, ages: 20–33 years, mean ± SD: 26.56 ± 4.5 years).

### 3.2 Somatosensory detection task

In this experiment we tested the effect of LGVS compared to the sham stimulation. Tactile threshold was estimated for the left index finger. During the somatosensory detection task, the beginning of the trial was signalled by an auditory cue. Each trial contained two 1200 ms intervals of time defined by a low tone, and separated by 500 ms. 600 ms after the tone either LGVS or sham stimulation started. LGVS or sham stimulation occurred in the first and second interval. Stimulation procedure and parameters were as Experiment 1. A near-threshold tactile stimulus was randomly delivered in the first or in the second interval. In half the trials there was no delay between the end of the LGVS/sham stimulation and the tactile stimulus, while in the other half a delay of 400 ms was introduced between the end of the GVS/sham stimulation and the tactile stimulus. This difference in the delay allowed us to investigate the time-course of vestibular-somatosensory interactions. Participants were divided in two groups according to the dose of GVS received. The duration of LGVS and sham stimulation was fixed at 100 ms in one group and at 200 ms in the other group. Participants were asked to detect a near-threshold tactile stimulus responding ‘first’ or ‘second’ interval. The task was divided into five different blocks; each block consists of sixteen trials. Participants were blindfolded and sat on a chair in a head-down posture.

### 3.3 Results

Because this was a two-alternative forced-choice experiment, we used percentage accuracy rather than signal detection analyses. A 2 Stimulation (LGVS vs sham stimulation) X 2 Delay (no delay vs 400 ms delay) within subjects and 2 Group (Low dose group or high dose group: 100 ms vs 200 ms) between subjects ANOVA was applied to rate of correct responses estimated for each experimental condition.

**Fig. 3:**
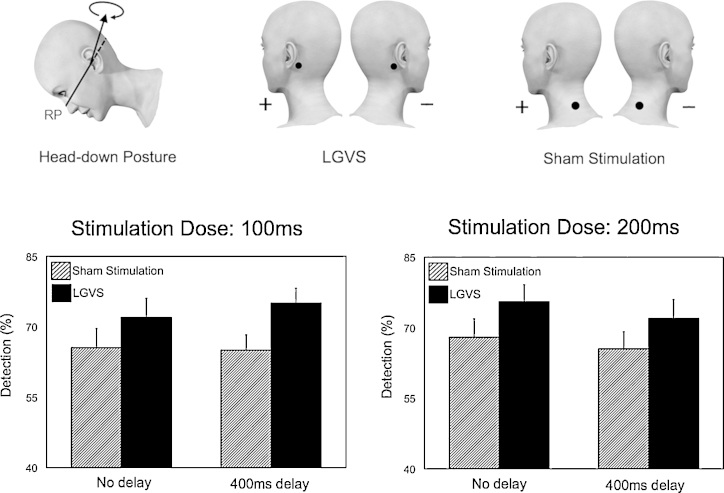
Effects of time and dose of LGVS and sham stimulation on somatosensory signal detection. Note the significant improvement of tactile detection induced by even minimal GVS. There were no differences between conditions involving different times and dose of GVS.

A highly significant main effect of Stimulation (*F*_(1,18)_= 9.415; *p* = 0.007) was found, with better performance during LGVS than during sham. No other significant effects or interactions were found (all *p* > 0.05). In particular, there was no effect of time between Stimulation and Delay (*F*_(1,18)_ = 0.055; *p* = 0.817), and no significant interaction between Stimulation and Group (*F*_(1,18)_ = 0.063; *p* = 0.804), suggesting no effect of LGVS dose (Fig. 3).

### 3.4 Discussion

Neither a significant time effect nor dose effect has been shown. Even minimal LGVS (1 mA, 100 ms) can alter somatosensory processing, inducing an enhancement of tactile perception. Therefore, the modulation of somatosensory detection is specifically due to vestibular activation, and induced even with small amount of stimulation.

## 4 General discussion

LGVS selectively improved the ability to discriminate faint tactile stimuli from the background noise, confirming previous findings obtained with CVS [4,6]. Additionally, this effect is clearly distinct from changes in response bias. The somatosensory enhancement induced by LGVS has been found for detection of shocks on both hands. Our data highlighted the specificity of this interaction since RGVS had no significant effect on tactile perception. Polarity-dependent GVS effects have been shown in brain-damaged patients [12,18] and in healthy participants [15]. Neuroimaging studies have identified an asymmetry in the cortical vestibular system, suggesting that the cortical vestibular network is primarily located in the non-dominant right hemisphere in right-handed subjects [1]. Therefore, the polarity-specific influence of LGVS on touch may be related to modulations of tactile processing in the right hemisphere. However, the mechanism that links GVS polarity effects to cortical dominance remains unclear. In particular, one might imagine that the dominant right hemisphere vestibular projections could be activated by both LGVS and RGVS [3,14,22], yet we found effects only of LGVS. However, fMRI studies identify a relatively stronger activation of the right hemisphere during LGVS compared to RGVS [7]. Thus, we cannot exclude that the right-hemispheric activation during RGVS was too weak to modulate the tactile processing.

One aim of our work was to investigate whether the tingling skin sensation evoked at GVS electrodes during stimulation could have acted as a cue for the participant. If so, GVS might change general arousal, or the attention to the cathodal side of the body where the tingling sensation is strongest. A sham stimulation was applied with same intensity, waveform and duration of GVS on the participant's neck, but did not induce any improvement in somatosensory sensitivity. Indeed, it non-significantly decreased the ability to detect faint tactile stimuli in both the fingers. We believe that our results clearly ruled out explanations based on somato-somatosensory interactions between the skin sensations evoked by vestibular stimulation, and also non-specific alerting effects.

Finally, our data suggest that even minimal amounts of GVS stimulation are enough to elicit the somatosensory modulation. The dose and latency of LGVS do not appear to influence the degree of somatosensory modulation, at least within the range studied here. We can speculate that the effect of LGVS operates at short latency, within 100 ms, to influence the somatosensory system. This speed is too rapid for plastic changes in synaptic connectivity, but instead suggests a model of direct integration of vestibular and somatosensory signals. In particular, the primary and secondary somatosensory cortex respond to both types of inputs, and are thus good candidates to subserve this integration. Functional responses suggest that area OP 2 is the key vestibular projection within PIVC. This area is localised within the Sylvian fissure at the junction of the posterior parietal operculum with the insular and retroinsular region [3]. OP 2 lies adjacent to area OP 1, which has been identified as the secondary somatosensory cortex (SII) in primates. We speculate that vestibular inputs could reach OP 2 in the non-dominant hemisphere, and there increase the firing of neurons that receive both tactile and vestibular inputs. This explanation also accounts for the sensitivity enhancement in both hands, since SII receives bilateral projections. Thus, GVS might induce excitatory postsynaptic potentials (EPSPs) in bimodal neurons, modulating their sensitivity to somatosensory inputs. However, excitatory post-synaptic effects are short-lived, so this hypothesis would not explain the modulation found several hundred milliseconds after the stimulation ended.

## 5 Conclusion

Brief galvanic vestibular stimulation enhanced somatosensory sensitivity on both hands. The effects were hemisphere-dependent: vestibular stimulation designed to activate the right hemisphere enhanced sensitivity, while vestibular stimulation designed to activate the left hemisphere did not. Successful interaction with the environment involves constant adjustment of multisensory inputs. The vestibular system may influence the processing within other individual sensory channels.

## Disclosure

All authors have approved the final article. There are no conflicts of interest for any author.
